# Efficacy of High-dose Liraglutide 3.0 mg in Patients with Poor Response to Bariatric Surgery: Real-world Experience and Updated Meta-analysis

**DOI:** 10.1007/s11695-023-07053-9

**Published:** 2024-01-06

**Authors:** Federica Vinciguerra, Carla Di Stefano, Roberto Baratta, Alfredo Pulvirenti, Giuseppe Mastrandrea, Luigi Piazza, Fabio Guccione, Giuseppe Navarra, Lucia Frittitta

**Affiliations:** 1 Department of Clinical and Experimental Medicine, University of Catania, Via Santa Sofia, 89, 95123 Catania, Italy; 2 General and Emergency Surgery Department, Garibaldi Hospital, 95122 Catania, Italy; 3 Endocrinology Unit, Garibaldi Hospital, 95122 Catania, Italy; 4 Bioinformatics Unit, Department of Clinical and Experimental Medicine, University of Catania, 95131 Catania, Italy; 5 Bariatric Surgery Unit, Candela Clinic, 90141 Palermo, Italy; 6 Department of Human Pathology, University of Messina, 98122 Messina, Italy; 7 Diabetes and Obesity Center, Garibaldi Hospital, 95122 Catania, Italy

**Keywords:** Liraglutide, Bariatric surgery, Insufficient weight loss, Weight regain

## Abstract

**Purpose:**

Poor response to bariatric surgery, characterized by insufficient weight loss (IWL) or weight regain (WR), poses a significant challenge in obesity treatment. This study aims to assess the effectiveness of liraglutide in addressing this issue.

**Materials and Methods:**

A retrospective, multicenter cohort study investigated the impact of liraglutide 3 mg on weight loss in adults with suboptimal responses or weight regain after bariatric surgery (BS). Additionally, a systematic review and meta-analysis were conducted for a comprehensive evaluation.

**Results:**

A total of 119 patients (mean age 41.03 ± 11.2 years, 71.4% female) who experienced IWL or WR after BS received pharmacologic therapy with liraglutide 3 mg. Mean percent weight loss in the entire cohort was 5.6 ± 2.6% at 12 weeks and 9.3 ± 3.6% at 24 weeks with a significant reduction in waist circumference (*p* < 0.0001). No serious side effects were reported. A meta-analysis, utilizing the fixed effect model with the metafor package in R, included 6 and 5 papers for the change in body weight and BMI after liraglutide treatment, respectively. The analysis demonstrated a considerable reduction in body weight (7.9; CI − 10.4; − 5.4, *p* < 0.0001) and BMI (3.09; CI 3.89; − 2.28, *p* < 0.0001).

**Conclusion:**

Liraglutide 3 mg emerges as a viable option for significant weight loss in patients experiencing IWL or WR after BS. Its inclusion in a multimodal, sequential obesity treatment approach proves promising.

**Graphical Abstract:**

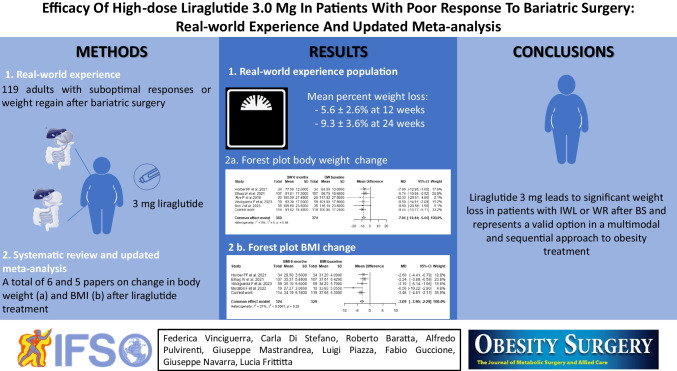

## Introduction

Bariatric surgery (BS) is the most effective treatment for people with severe obesity, resulting in significant and sustained weight loss, improved quality of life, improvement or remission of obesity-related comorbidities, and a reduction in cardiovascular events, cardiovascular deaths, and all-cause mortality [1–4]. Nevertheless, the response to BS varies greatly at the individual level for reasons that are partly unknown. Individuals who respond poorly to BS may experience insufficient weight loss (IWL) or clinically relevant weight regain (WR) after a successful primary intervention [5], which may compromise the overall benefits of weight loss, favor the persistence or recurrence of comorbidities, and worsen the quality of life [6–9]. The prevalence of WR or IWL is difficult to estimate as there are no clear definitions [10–15], and the treatment of these conditions is still unclear. Surgical treatment (i.e., revision of the gastric pouch and anastomosis or conversion to a more effective procedure) is a valid option that should only be performed in experienced BS centers due to the higher complexity and comorbidities compared to primary surgery [16]. Recently, the ketogenic diet has been described as an effective and safe short-term strategy for the treatment of poor response to BS [17]. However, there is an unmet clinical and non-surgical need for effective therapeutic options beyond the nutritional and psychological approach [18]. The use of pharmacotherapy for weight loss, indicated in addition to lifestyle interventions for adult patients with a BMI ≥ 30 kg/m2 or in subjects with BMI ≥ 27 kg/m2 but with at least one weight-related comorbidity [19, 20], generally results in greater weight loss and maintenance. Liraglutide 3.0 mg is the first GLP-1 analog approved for this therapeutic indication and may represent an available option for the treatment of patients who respond poorly to BS [18]. A randomized clinical trial [21] and several observational studies [22–26] have investigated whether this pharmacotherapy could benefit people with low post-bariatric weight loss. Following our initial observational study of liraglutide 3.0 mg, which resulted in improvement in metabolic syndrome [27], we have extended our clinical experience and, to our knowledge, conducted the first meta-analysis to evaluate the efficacy of this anti-obesity drug in the treatment of patients with poor response to BS.

## Materials and Methods

### Original Data

This is a retrospective observational study including patients referred to our obesity center aged 18 to 65 years who received liraglutide as pharmacological therapy for the treatment of WR or IWL after BS. Patients underwent restrictive procedures (adjustable gastric banding or sleeve gastrectomy) or malabsorptive procedures (Roux-en-Y gastric bypass or one-anastomosis gastric bypass). IWL was defined as an initial weight loss of less than 50% of the excess weight loss (EWL), WR was defined as a weight gain of at least 15% of the weight lost after bariatric surgery [28]. WR was calculated using the following formula: (current weight − nadir weight)/ (pre-bariatric weight − nadir weight) × 100 [15].

Demographic, anthropometric, and clinical data were collected retrospectively from the patients’ electronic records.

Exclusion criteria were the concurrent use of other weight-loss medications, pregnancy or breastfeeding, acute and/or severe organ disease, type 1 or 2 diabetes, congestive heart failure (NYHA class IV), a personal or family history of medullary thyroid carcinoma (MTC) or multiple endocrine neoplasia type 2 (MEN2), a personal history of non-familial MTC, and a personal history of acute or chronic pancreatitis.

The Catania 2 Ethics Committee approved the study (94/CECT2, 27/09/2022). The study was conducted in accordance with the principles of the Declaration of Helsinki.

### Systematic Review and Meta-analysis

The systematic review was conducted according to MOOSE (Meta-analysis Of Observational Studies in Epidemiology) guidelines [29].

The search was performed by two independent authors in the online databases PubMed and Scopus, and the references of the included studies were checked to find further papers. The following terms were used in the search: (“liraglutide” or “GLP-1 RA”) and (“weight-regain” and/or “insufficient weight loss” and/or “poor-response” and/or “bariatric surgery”). The last search was conducted on September 23, 2023. The two reviewers independently screened the papers, reviewed both titles and abstracts, reviewed the full texts, and selected articles for inclusion.

Inclusion criteria were English-language studies, without restriction of publication year, that reported original data on body weight and/or BMI, before and after at least 6 months of treatment with liraglutide 3 mg for the treatment of WR or IWL after bariatric surgery.

All data were extracted from the main text, tables, figures, and supplementary material of the studies.

If relevant data were missing, the corresponding authors were asked to provide them. Only the studies for which we received all data were included in the meta-analysis.

The risk of bias of the included studies was assessed by two reviewers using the National Heart, Lung, and Blood Institute Quality Assessment Tool for Observational Studies (*National Heart, Lung, and Blood Institute. Study quality Assessment Tools* (*2023*)*. *https://www.nhlbi.nih.gov/health-topics/study-quality-assessment-tools).

### Statistical Analysis

Continuous variables are described as mean ± standard deviation (SD), while categorical variables are expressed as number and percentage. All continuous variables were normally distributed (Kolmogorov–Smirnov test). Longitudinal changes were assessed using the paired Student’s *t*-test. For categorical variables, statistical significance between groups was determined using the chi-square test. The sample size was calculated based on a previous study (27). The significance threshold was set at *p*-values of < 0.05. Data analysis was performed using STATA/SE 17.0.

We performed a meta-analysis with the fixed effect mode using the metafor package of the R system.

The meta-analysis was performed in the group of patients at baseline compared to the same patients at 6 months on liraglutide. Since we had the same scale for the outcome variable at baseline and 6 months, we used the mean difference (MD) of body weight (first meta-analysis) and BMI (second meta-analysis).

## Results

The sample consisted of 119 patients with a mean age of 41.0 ± 11.2 years. Most patients were female (71.4%, *n* = 85). The entire population had a mean body weight of 100.9 ± 17.2 kg and a mean BMI of 37.6 ± 5.3 kg/m^2^, with no difference between genders.

Patients who underwent malabsorptive procedures (*n* = 37) had a higher body weight (108.2 ± 15.5 vs 97.7 ± 17.1, *p* = 0.001) and BMI (40.2 ± 5.3 vs 36.5 ± 4.9, *p* = 0.0004) than patients who underwent restrictive BS (*n* = 82).

The clinical characteristics at baseline and after treatment are shown in Table 1.

**Table 1:** Clinical parameters at baseline and after 12 and 24 weeks of treatment in our population

Clinical parameters	Baseline	After 12 weeks	After 24 weeks
Body weight (kg)	100.9 ± 17.2	95.3 ± 16.8*	91.5 ± 16.49*
BMI (kg/m^2^)	37.6 ± 5.3	35.6 ± 5.3*	34.2 ± 5.2*
WC (cm)	118.8 ± 13.3	114.5 ± 12.8*	110.5 ± 12.8*

^*^p < 0.0001 vs baseline

After 12 weeks of treatment with liraglutide, the mean percent weight loss in the entire cohort was 5.6% ± 2.6% with a significant reduction in waist circumference (WC) (118.8 ± 13.3 vs. 114.5 ± 12.8, *p* < 0.0001). At this time point, patients who underwent restrictive procedures achieved significantly greater weight loss than patients who underwent malabsorptive procedures (WL% 6.01 ± 2.7 vs. 4.7 ± 2.3, *p* < 0.01).

After 12 weeks of treatment with liraglutide, most patients (*n* = 67, 56.3%) lost at least 5% of their body weight: 43.7% (*n* = 52) lost between 0 and 5%, 50.4% (*n* = 60) between 5 and 10%, and 5.8% (*n* = 7) more than 10% of their body weight.

After 24 weeks, the mean percentage weight loss was 9.3 ± 3.6% with a significant reduction in WC (− 8.7 ± 5.5, *p* < 0.0001). No significant differences according to BS procedures were found.

Most patients (*n* = 107, 93.8%) lost at least 5% of their body weight: 6% (*n* = 7) lost between 0 and 5%, 59.6% (*n* = 68) lost between 5 and 10%, 27% (*n* = 31) lost between 10 and 15%, and 7% (*n* = 8) lost more than 15% of their body weight.

## Meta-analysis

### Body Weight

**Fig. 1:**
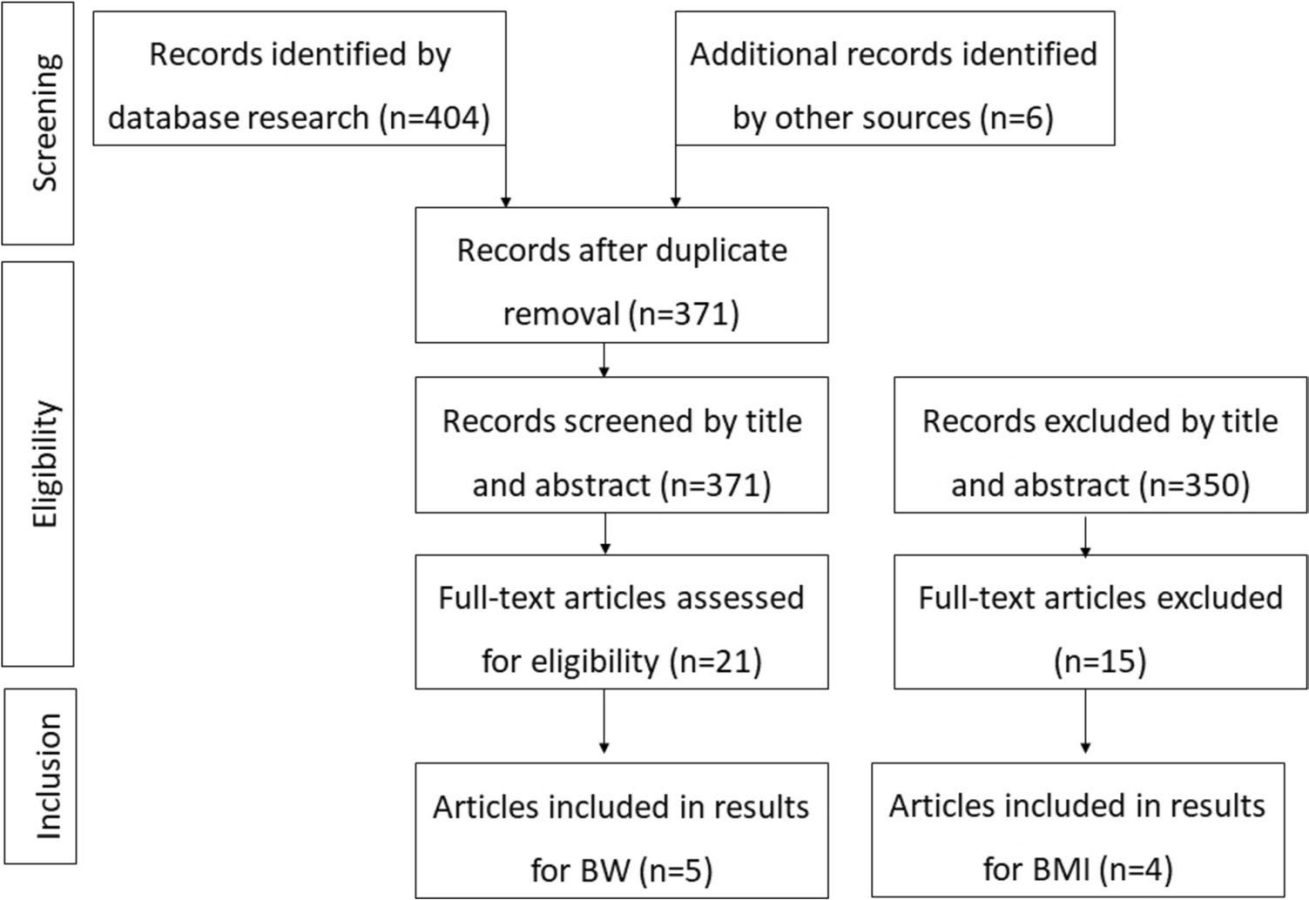
Flow of records found

A total of 5 papers published between 2018 and 2023 were included in the meta-analysis on the change in body weight after liraglutide treatment [21, 24–27] (Fig. 1). Our current work was added. All papers were retrospective, with the exception of the paper by Horber, a prospective study comparing the effects of liraglutide treatment with revision surgery, and the study by Mok, the only randomized clinical trial.

**Fig. 2:**
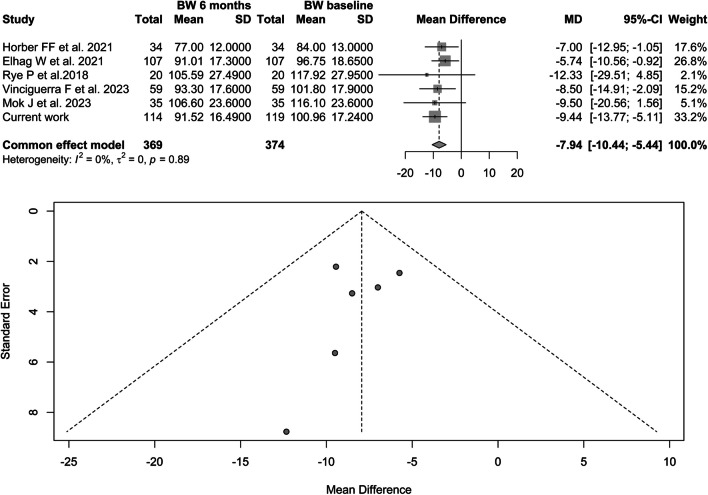
Forest plot body weight before and after 24 weeks of liraglutide therapy

A total of 369 patients were treated in the selected studies (Fig. 2). The main characteristics of the studies and the clinicopathological characteristics of the patients included in the meta-analysis are summarized in Table 2.

**Table 2:** Main characteristics of the studies included in the meta-analysis

First author	Year	Study design	Sample size	Type of BS	Poor response	Liraglutide mean dosage
Horber FF et al	2021	Prospective	34	RYGB	WR	2 mg
Elhag W et al	2021	Retrospective	107	AGB, SG, RYGB	WR, IWL	3 mg
Rye P et al	2018	Retrospective	20	AGB, SG, RYGB, VBG	WR, IWL	2.9 mg
Vinciguerra F et al	2023	Retrospective	59	SG, OAGB	WR, IWL	2.4 mg
Mok J et al	2023	RCT	35	SG, RYGB	IWL	3 mg
Muratori F et al	2022	Retrospective	10	AGB, SG, RYGB	WR	3 mg

*RCT* randomized controlled trial, *RYGB* Roux-en-Y gastric bypass, *AGB* adjustable gastric band, *SG* sleeve gastrectomy, *VBG* vertical banded gastroplasty, *OAGB* one-anastomosis gastric bypass

### Body Mass Index (BMI)

**Fig. 3:**
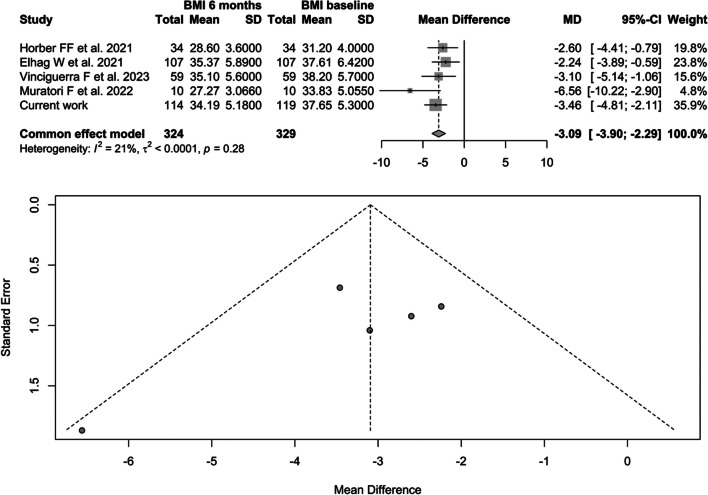
Forest plot BMI before and after 24 weeks of liraglutide therapy

The meta-analysis on the change in BMI after 24 weeks included 3 retrospective [23, 25, 27] and only 1 prospective study [24] (Table 2). In total, the selected studies included 324 patients (Fig. 3).

## Discussion

This retrospective study, conducted in 119 patients with poor response to bariatric surgery, shows that high-dose liraglutide is an effective treatment for weight loss. After 3 months of treatment, 50% of patients achieved a weight loss of between 5 and 10%. After 24 weeks of treatment, a weight loss of over 9% was achieved, combined with a significant reduction in WC (Table 1).

In our sample, patients undergoing restrictive procedures achieved greater weight loss at baseline, after 12 weeks, than patients undergoing malabsorptive procedures, unless they had a lower baseline BMI; however, no differences were observed after 24 weeks (Fig. 3). There is no evidence in the literature of a differential response to pharmacotherapy depending on the type of procedure, although most studies evaluate efficacy after at least 24 weeks [22, 23, 27]. One of the main problems for poor response to BS in restrictive surgery is the reduction in satiety and progressive increase in gastric capacity, which are the main mechanism of action of the procedure. In this context, liraglutide seems to act directly in hypothalamic areas, improving satiety stimulus and reducing hunger sensation but also slowing down gastric emptying peripherally [30], partially restoring the original mechanism of the restrictive BS procedure.

In our cohort of patients, no difference in weight loss was observed regardless of the type of poor response (IWL or WR), and these results are similar to those previously reported [27].

Despite anatomical and physiological changes after surgery that could favor gastrointestinal discomfort, treatment with liraglutide in our population had no side effects different from those reported in non-bariatric patients. The discontinuation of treatment after 12 weeks was solely due to the high cost of the drug, which is not reimbursable in our country.

Therefore, our data suggest that liraglutide is an effective option for the treatment of patients who respond poorly to bariatric surgery; however, there is still no clear indication in the guidelines. We conducted a systematic review and meta-analysis to assess and summarize the evidence on the efficacy of liraglutide 3.0 mg from all available data. The current meta-analysis showed a reduction in body weight of 7.9 (CI − 10.4; − 5.4, *p*-value < 0.0001) and a reduction in BMI of 3.09 (CI 3.89; − 2.28, *p*-value < 0.0001).

Although some studies could not be included due to missing or heterogeneous data and the studies analyzed were mainly observational and retrospective studies, the meta-analysis showed that pharmacotherapy with liraglutide has a positive effect on weight loss.

Another possible and effective solution for patients who do not respond adequately to bariatric surgery is revision surgery. Horber et al. [24] have shown that pharmacotherapy with liraglutide is similarly effective in reversing weight gain as revision surgery to restore restriction (endo-surgery or implantation of a Fobi ring), with a lower complication rate. Re-intervention after BS is known to be associated with a relatively high complication rate, and liraglutide could be a first-line treatment for poor response, while revision surgery could be considered for patients who do not respond to liraglutide treatment. In addition, Elhag reported that liraglutide 3.0 mg was equally effective in managing modest weight loss after primary or revision metabolic surgery, with no difference in side effects [25].

In the absence of specific guidelines for medical weight management in poor responders to BS, the results of the current study and meta-analysis provide data to support clinicians in the use of liraglutide to treat such patients.

Obesity is a complex, chronic, relapsing disease, and there is no universally effective method that achieves the same results in all patients. To overcome these limitations, a lifelong, multimodal combined approach that includes cognitive-behavioral, nutritional, pharmacological, and surgical therapy should be proposed for all patients with obesity.

## Conclusion

Our research supports the efficacy of liraglutide in facilitating weight loss in individuals with IWL or WR after BS. Over a 12-week period, liraglutide 3 mg was shown to result in an average weight loss of 5.6%, associated with a significant reduction in waist circumference. Our pivotal meta-analysis, the first to evaluate the efficacy of liraglutide in patients with a suboptimal response to BS, corroborated our study, showing a 5% body weight loss in 93.8% of cases. These results provide valuable insight into the multiple benefits of liraglutide in the treatment of obesity and warrant further investigation into optimal treatment duration and dosage.

## Data Availability

The raw data supporting the conclusions of this article will be made available by the authors without undue reservation.
